# *In vitro* modeling of the neurobiological effects of glucocorticoids: A review

**DOI:** 10.1016/j.ynstr.2023.100530

**Published:** 2023-02-23

**Authors:** Katherine Bassil, Anthi C. Krontira, Thomas Leroy, Alana I.H. Escoto, Clara Snijders, Cameron D. Pernia, R. Jeroen Pasterkamp, Laurence de Nijs, Daniel van den Hove, Gunter Kenis, Marco P. Boks, Krishna Vadodaria, Nikolaos P. Daskalakis, Elisabeth B. Binder, Bart P.F. Rutten

**Affiliations:** a Department of Psychiatry and Neuropsychology, School for Mental Health and Neuroscience (MHeNs), Faculty of Health, Medicine and Life Sciences, Maastricht University, Maastricht, the Netherlands; b Department of Translational Research in Psychiatry, Max Planck Institute of Psychiatry, Munich, Germany; c Institute of Neuroscience, Université Catholique de Louvain, Brussels, Belgium; d Department of Psychiatry, McLean Hospital, Harvard Medical School, Belmont, MA, USA; e Department of Translational Neuroscience, University Medical Center (UMC) Utrecht Brain Center, Utrecht University, Utrecht, the Netherlands; f Psychiatry, UMC Utrecht Brain Center, Utrecht, the Netherlands; g Salk Institute for Biological Studies, La Jolla, San Diego, United States; h International Max Planck Research School for Translational Psychiatry, Munich, Germany

**Keywords:** Stress, Stress disorders, Glucocorticoids, In vitro models, Psychiatry, Neurobiology

## Abstract

Hypothalamic-pituitary adrenal (HPA)axis dysregulation has long been implicated in stress-related disorders such as major depression and post-traumatic stress disorder. Glucocorticoids (GCs) are released from the adrenal glands as a result of HPA-axis activation. The release of GCs is implicated with several neurobiological changes that are associated with negative consequences of chronic stress and the onset and course of psychiatric disorders. Investigating the underlying neurobiological effects of GCs may help to better understand the pathophysiology of stress-related psychiatric disorders. GCs impact a plethora of neuronal processes at the genetic, epigenetic, cellular, and molecular levels. Given the scarcity and difficulty in accessing human brain samples, 2D and 3D *in vitro* neuronal cultures are becoming increasingly useful in studying GC effects. In this review, we provide an overview of *in vitro* studies investigating the effects of GCs on key neuronal processes such as proliferation and survival of progenitor cells, neurogenesis, synaptic plasticity, neuronal activity, inflammation, genetic vulnerability, and epigenetic alterations. Finally, we discuss the challenges in the field and offer suggestions for improving the use of *in vitro* models to investigate GC effects.

## 1 Introduction

### 1.1 Stress and stress-related disorders

Stress can be defined as any change to the environment, either internal or external, that may lead to homeostatic disruption or imbalance. This definition takes into account variations that may accompany individual stress responses and disparate effects of a single stress stimulus (Leonard, 2005a). The relationship between stress and ill-health is not straightforward. Stressors can elicit various responses depending on a number of factors that include, but are not limited to sex, developmental time-window of the exposure, genetics and type and length of the stressor. For example, acute stress has been shown to enhance brain and physical functioning while chronic stress can often lead to severe illnesses, both behavioural and physical (Salleh, 2008). A stressor is defined as a physical and or psychological stimulus that disturbs homeostasis and activates a stress response aimed at restoring a state of balance while preparing for potential future stressors. In case of persistent or chronic exposure to a stressor the adaptive responses of an organism can become exhausted, creating a new non-functional balance (Del Giudice et al., 2011; Ramsay and Woods, 2014; Sterling, 1988; McEwen and Stellar, 1993; McEwen and Wingfield, 2010; Schulkin, 2011; Olff et al., 2005; Jezova et al., 2016), which has been linked to increased risk for a range of stress-related disorders (SRDs) such as major depressive disorder (MDD) and post-traumatic stress disorder (PTSD) (Selye, 1936). The group of SRDs thus refers to disorders that can be characterized by maladaptive responses to traumatic or stressful event(s) in a given period of time (Organization, 1992).

While evidence supports a strong role for exposures to chronic or severe stress and/or trauma in the aetiopathogenesis of psychiatric and physical disorders, it has also been noted that not all individuals will suffer the consequences of chronic stress. Instead, a considerable proportion of individuals show tolerance to stressful or traumatic situations. Clinically, the latter is referred to as the phenomenon of resilience, while individuals that display a maladaptive stress response are referred to as being vulnerable or susceptible to stress (Karatsoreos and McEwen, 2011).

### 1.2 The stress response

**Fig. 1:**
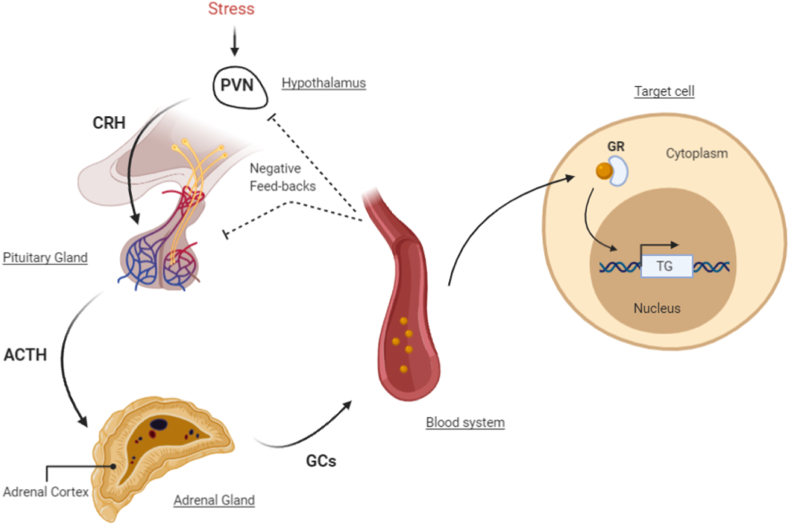
Stress activation of the hypothalamic-pituitary adrenal (HPA) axis. After exposure to a stressful situation, the activity of the HPA axis is increased. In those conditions, the paraventricular nucleus (PVN) releases corticotropin-releasing hormone (CRH). CRH then binds to its receptor in the anterior part of the pituitary gland promoting the secretion of adrenocorticotrophic hormone (ACTH) into circulation. Finally, ACTH reaches the adrenal gland and stimulates the production of glucocorticoids (GC) by the adrenal cortex of the adrenal glands. Therefore, GCs will be secreted into the bloodstream and reach diverse cells and organs in the body, leading to the transcription of target genes via activation of glucocorticoid receptors (GR). As a part of homeostatic mechanisms in the body, the HPA axis is subject to robust negative feedback inhibition by GCs (This figure has been created with BioRender.com).

The primary stress-response systems in mammals are the sympathetic nervous system and the hypothalamic-pituitary adrenal (HPA)-axis (Charmandari et al., 2005). Glucocorticoids (GCs) are predominantly released by the HPA-axis and are key elements in the first response to a stressor as well as in the long-term physiological responses to stress (Fig. 1) (Nicolaides et al., 2015). In brief, during a stressful event, parvocellular neurons of the paraventricular nucleus (PVN) of the hypothalamus secrete corticotropin-releasing hormone (CRH) in the venous portal system of the pituitary. In the anterior pituitary, CRH stimulates corticotropic cells to synthesize adrenocorticotropic hormone (ACTH), which is released in the blood stream. In turn, ACTH stimulates the production and secretion of GCs, which are steroid hormones, from the adrenal cortex (Herman et al., 2012a). In humans the main endogenous GC is cortisol (CORT) whereas in rodents it is corticosterone. The pulsatile release of GCs follows a circadian and ultradian rhythm which results in peak levels of GCs in the mornings (Spiga et al., 2014). Circulating GCs are related to a plethora of physiological processes such as energy mobilization, metabolic changes, and immune responses. During acute stress, HPA-axis activity is rapidly increased which leads to higher levels of circulating GCs (Russell and Lightman, 2019). Stress-induced GC levels in pathological states (between 420 and 779 nM (Davis et al., 1981)) have been shown to be several fold higher than diurnal baseline levels of circulating GC levels (between 137 and 283 nM) (Pirich and Vierhapper, 1988)) (Cay et al., 2018; Elzinga et al., 2003). The effects of GCs are mediated by two types of steroid receptors: the glucocorticoid receptor (GR), encoded by the nuclear receptor subfamily 3 group C member 1 (*NR3C1)*, and the mineralocorticoid receptor (MR), encoded by the nuclear receptor subfamily 3 group C member 2 (*NR3C2),* with endogenous GCs harbouring higher affinity to the MR than the GR (Russell and Lightman, 2019; De Kloet, 2000).

Following the binding of GCs, the receptor is activated and may induce both genomic and non-genomic pathways. Focusing on the genomic pathway, the activated receptor translocates to the nucleus and acts as transcription factor by binding to specific DNA sequences known as glucocorticoid response elements (GREs) (Grad and Picard, 2007). These GREs influence the transcriptional expression of genes (Lightman et al., 2020) involved in numerous physiological processes such as inflammation (acting as anti- or pro-inflammatory facilitator) (Cruz-Topete and Cidlowski, 2015), synaptic plasticity (Madalena and Lerch, 2017), and apoptosis (Almeida et al., 2000).

Under normal circumstances, once the stressor subsides, the HPA-axis is dampened via the inhibiting effects of GCs at the level of the PVN and the pituitary. This negative-feedback mechanism relies heavily on GC-GR signalling (Herman et al., 2012b). A key player in the regulation of GR expression is FKBP prolyl isomerase 5 (*FKBP5*), acting as a co-chaperone to the GR influencing its sensitivity to GCs (Binder, 2009). Increasing evidence points towards dysregulation of the neuroendocrine system in subsets of patients with PTSD (Speer et al., 2019) and MDD (Lopez-Duran et al., 2009), predominantly within the HPA-axis (Connor and Davidson, 1998; Jones and Moller, 2011), even though these are not always consistent. HPA-axis dysregulation can be measured with the dexamethasone (DEX) suppression tests (DST). DEX is a synthetic glucocorticoid and selective GR agonist, that, when administered, stimulates the negative feedback loop resulting in suppression of GC release. DST studies suggest that the HPA-axis may be hypo-suppressed in MDD and hyper-suppressed in PTSD (Daskalakis et al., 2016). However, it remains unclear whether this HPA-axis dysregulation is a cause, consequence, mediator, or moderator in the development of SRDs (Klaassens, 2010; Aerni et al., 2004, de Quervain, 2008; Buitelaar, 2013). It should also be noted that HPA-axis dysregulation is mainly reported in conditions of early life adversity, implying a neurodevelopmental context for SRD pathogenesis (Heim et al., 2008).

### *1.3* *In vitro* brain models

Given the scarcity and difficulty in the use of human brain tissue as well as the ethical implications associated with it, scientists have turned to animal and cellular models in order to better understand how GCs contribute to stress reactivity and neurobiological changes (Tonhajzerova and Mestanik, 2017). Animal models have indisputable importance for the study of the brain at physiological and disease conditions as well as in response to environmental stimuli. This review will focus on *in vitro* models used as an additional way to study aspects of brain functioning.

Despite the limitations of *in vitro* studies, they have regained attention in the past decade, especially through the advent of induced pluripotent stem cell (iPSC)-derived models, which allow the direct investigation of patient-derived cells and disease-specific phenotypes. These models are now being considered as one of the pivotal pillars of contemporary neurobiology research due to their numerous advantages. In addition to the possibility of generating cells of human origin, other advantages of iPSC-derived models include the potential for straightforward drug testing, genetic and epigenetic manipulations, and relatively lower costs than *in vivo* experiments. Moreover, the need for robust *in vitro* model systems is warranted by increasing international efforts founded on the 3R principle (Refining, Reducing, and Replacing animal models) for animal research (Jonsson et al., 2016). Therefore, combining *in vivo* and *in vitro* studies to explore certain mechanisms is vital.

**Fig. 2:**
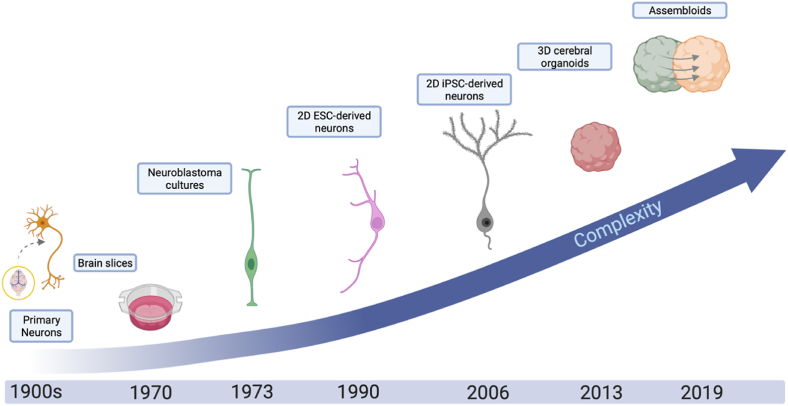
Evolution of ***in vitro* brain models used for investigating effects of glucocorticoids**. Schematic representation of past and emerging *in vitro* neuronal models with increasing resemblance to human *in vivo* brain functioning, that have been used for the investigation of the neurobiological effects of glucocorticoids. These models include primary neurons, brain slices and neuronal networks (e.g., organotypic slice cultures), neuroblastoma cultures, 2D pluripotent-stem cell-derived (PSC) neurons, 3D organoids of different brain-regions and assembloids (This figure has been created with BioRender.com).

**Fig. 3:**
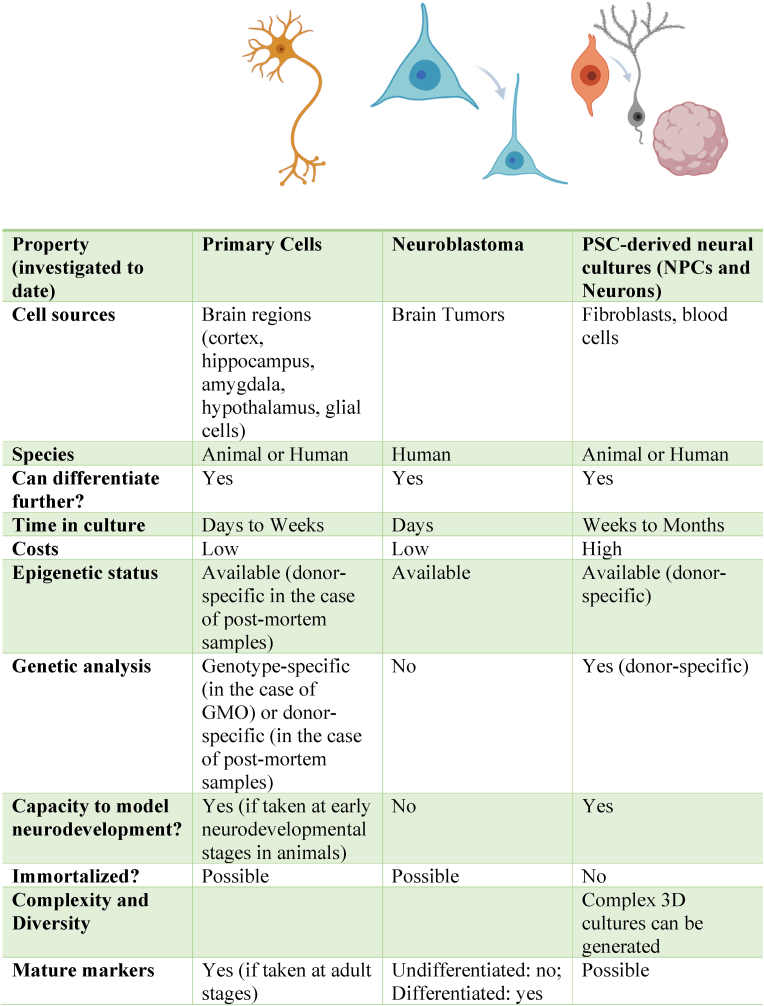
Comparing different *in vitro* brain models used for investigating neurobiological effects of glucocorticoids (Images have been created with BioRender.com) Abbreviations: PSC, pluripotent stem cells; NPC, neural progenitor cells; GMO, genetically modified organism.

A variety of *in vitro* neuronal models have been used to investigate the effects of GCs on neuronal processes. These range from animal primary neuronal cultures, *ex-vivo* brain slices*,* animal or human neuroblastoma cell lines (e.g., SH-SY5Y cells) and embryonic stem cell (ESC)- or iPSC-derived neuronal models. These include both 2-dimensional (2D) cultures and 3-dimensional (3D) organoid cultures that model certain brain regions, such as the cortex or the hippocampus (Fig. 2). The efforts to model more than one brain region are now focusing on combining organoids of different regions in one structure called assembloids (Vogt, 2021; Huang, 2021; Sloan et al., 2018; Bagley et al., 2017). Each of these models can be used to answer specific research questions and each carries its unique advantages and disadvantages (Fig. 3). For instance, primary neuronal cultures and *ex-vivo* brain slices maintain high fidelity to *in vivo* biology but are a less abundant resource. Neuroblastoma cell lines hold a relatively lower cost compared to primary cultures and carry human-specific biology which can be missing in rodent cultures. Additionally, they can be used both in their immature-undifferentiated stage as well as at a more mature-differentiation stage (Kovalevich and Langford, 2013). On the other hand, these cells are cancerous and have been genetically modified to induce stable, proliferating cultures and do not recapitulate the physiological proliferation, maturation, and death cycles of neuronal cells.

**Fig. 4:**
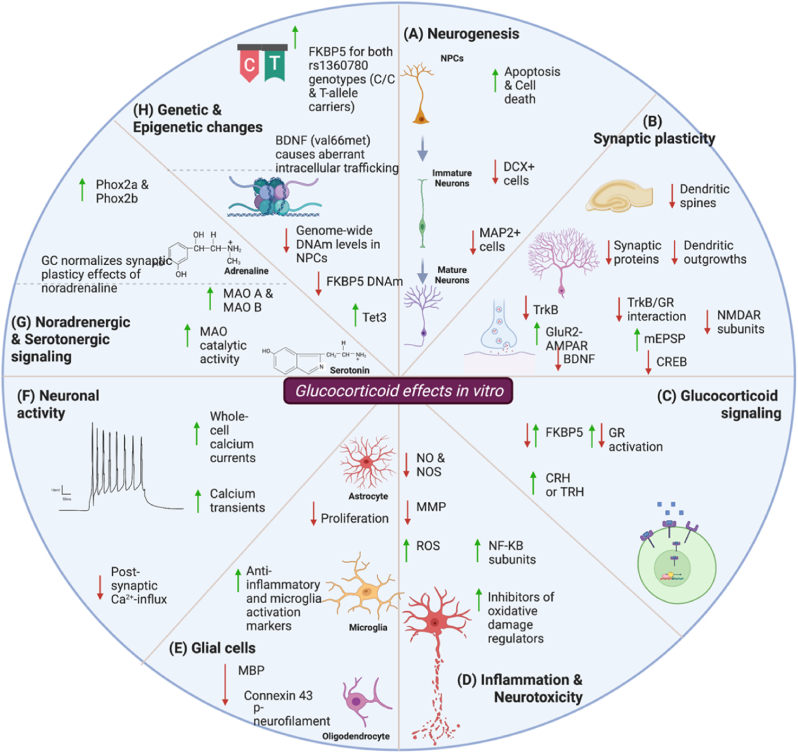
**Key findings of studies investigating glucocorticoid's neurobiological effects *in vitro*** Exposure to GCs affects many neurobiological aspects, including neurogenesis, GC signalling, inflammation and toxicity, myelination, synaptic plasticity, physiological activity, and genetic and epigenetic mechanisms. (A) GCs impact neurogenesis by having an effect on neural progenitor proliferation and survival, and decreasing the process of generating new neurons. (B) GCs negatively impacts synaptic plasticity particularly in hippocampal neurons by downregulating essential synaptic proteins, dendritic spines and outgrowths. (C) GCs alter glucocorticoid signaling and result in a downregulation of GR activity and translocation. (D) GC exposure exhibits both anti- and pro-inflammatory properties with the latter leading to an increase in neurotoxicity markers such as reactive oxygen species. (E) GCs alter glial functioning by decreasing the levels of myelin-associated proteins, proliferation of astrocytes, and increasing microglia activation markers. (F) GCs alter neuronal activity as seen with increases in calcium transients and currents. (G) Changes in noradrenergic and serotonergic signaling following GC administration. (H) Changes in epigenetic mechanisms, particularly DNA methylation, has been observed following GC exposure, possibly impacting the function of several regulatory genes, such as FKBP5. GC exposure leads to an increase in the GR regulator *FKBP5* in individuals carrying the *FKBP5* variant rs1360780. Abbreviations: BDNF, brain-derived neurotrophic factor; CRH, corticotrophin-releasing hormone; CREB, cAMP response element-binding protein; DCX, doublecortin; FKBP5, FK506 binding protein 5; GC, glucocorticoid; GluR2-AMPAR, GluR2 subunit-AMPA receptor; GR, glucocorticoid receptor; MAP2, microtubule-associated protein 2; mEPSP, miniature excitatory postsynaptic potential; MBP, myelin binding protein; MMP, mitochondrial membrane potential; MAO monoamine oxidase; NPCs, neural progenitor cells; NF-kB, nuclear factor kappa B; NO, nitric oxide; NOS, nitric oxide synthase; ROS, reactive oxygen species; NMDAR, N-methyl-D-aspartate receptor; TRH, thyrotropin-releasing hormone; TrkB, tropomyosin receptor kinase B (This figure has been created with BioRender.com).

This review outlines recent findings on some of the molecular and cellular mechanisms underlying GC effects *in vitro*, which can provide some evidence for mechanisms involved in susceptibility to SRDs (Faye et al., 2018; McEwen et al., 2015). We do acknowledge that the neurobiology of stress does not rely solely on the effects of GCs, and that GC exposure does not translate to stress exposure *in vitro* (MacDougall-Shackleton et al., 2019). For instance, noradrenaline, CRH, and other stress-related hormones all play a critical role in the stress response. And the effects of GCs only partially explain the stress response and its effects on cells in the central nervous system (CNS) and the development of SRDs. Additionally, inducing cellular stress mechanisms *in vitro* can be performed beyond treating cells with GCs, and that includes models of oxidative stress, nutrient deprivation, heat shock, treatment with chemicals (e.g., toxins), and mechanical stress, among others (Boks et al., 2018). However, as the literature on this topic is quite expansive in relation to SRDs, we provide an overview of a selected number of critical landmark studies (as opposed to providing a systematic review of the available literature). We start by mentioning limitations and challenges within the field such as the difficulty of identifying and optimizing experimental conditions and outcome parameters to differentiate between adaptive (allostasis) and maladaptive (allostatic load) responses, and the differential effects of acute versus chronic stress *in vitro*. We review studies that make use of GCs (namely CORT; corticosterone in animals or cortisol and hydrocortisone in humans, and DEX) because of their key role in the stress response and in stress susceptibility (Vyas et al., 2016). We focus on some of the most commonly used *in vitro* models and approaches the field is advancing. We begin by highlighting findings involving genetic liability/moderation and epigenetic changes following GC exposure. We then discuss GC-induced effects on molecular and cellular processes including neurogenesis, synaptic plasticity, and neurotoxicity among others. We end by highlighting studies looking into the effects of GCs on neurotransmitter systems and glial cells. The findings of the studies are described and summarized in Table 1, together with an overview highlighting some key findings in Fig. 4. Finally, we provide future perspectives on the importance of developing better *in vitro* models for investigating the neurobiological effects of GCs.

**Table 1:** *In vitro* studies examining the role of glucocorticoids in central nervous system cell lines.

a. Genetic and epigenetic variations underlying GC effects					
Publication	Cell line/model	Glucocorticoid tested	Concentration(s) used	Exposure duration	Primary finding
(Lieberman et al., 2017)	Human iPSC-derived neurons	dexamethasone	1 μM	6 h	DEX exposure leads to an increase in FKBP5 mRNA expression regardless of the FKBP5 genotype.
(Nold et al., 2021)	Primary mouse astrocytes, microglia, and (cortical and hippocampal) neurons	Dexamethasone or corticosterone	0.8, 4, 20, and 100 nM	4 h	Astrocytes, microglia, and neurons exhibit differential *FKBP5* expression in response to GCs, with astrocytes being the most responsive. These results further highlight the role of astrocytes in the stress response and *FKBP5-*associated functions.
(Seah et al., 2022)	Human iPSC-derived mixed forebrain neurons and induced-NGN2 neurons	dexamethasone and hydrocortisone	100, 1000, and 2500 nM	6 and 24 h	GC exposure produces cell-type specific stress responses and concentration-dependent differential genetic expression that could be used as a diagnostic tool for PTSD risk.
(Hay et al., 2014)	Primary neonate neurons	dexamethasone	50 μM	16 h	SNPGR, is a DEX response element of the TAC1 (gene encoding substance-P) promoter region which leads to an increased promoter activity if carrying the T-allele.
	SH-SY5Y neuroblastoma	dexamethasone	50 μM	24 h	SNPGR, is a DEX response element of the TAC1 (gene encoding substance-P) promoter region which leads to an increased promoter activity if carrying the T-allele.
(Bose et al., 2010)	Sprague Dawley Rat embryonic neural stem cells [E15]	dexamethasone	1 μM	48 h	DEX exposure reduces proliferation of NSC, upregulates genes associated with cellular senescence, and downregulates genes related to mitochondrial functions, possibly due to changes in gene methylation and leading to increased vulnerability to oxidative stress in daughter cells.
(Bose et al., 2015)	Sprague-Dawley rat primary cortical neural stem cell cultures [E15]	dexamethasone	1 μM	48 h	DEX exposure led to a genome wide hypomethylation associated with a decrease in Dnmt3a and an increase in Dkk1 via an increase in Tet3 expression.
(Provençal et al., 2020)	Hippocampal progenitor cells and neurons	dexamethasone	1 μM	3 or 10 days	Changes in DNAm and RNA expression followed DEX exposure. These changes were enhanced at human brain fetal development stages. Long lasting DMSs correlated with a second acute GC exposure.
(Lee et al., 2010)	Mouse HT22 hippocampal neurons	corticosterone	1 μM	6 h, 1; 3; 5; 7 days (with and without washout for 7 days)	Following chronic CORT exposure an increase in FKBP5 mRNA expression was accompanied by a decrease in DNA methylation.

b. Molecular underpinnings of GC effects					
Publication	Cell line/model	Glucocorticoid tested	Concentration(s) used	Exposure duration	Primary finding
(Verjee et al., 2018)	SH-SY5Y neuroblastoma	dexamethasone	10 μM	6 or 48 h	Both short and long DEX exposure led to an increase in FKBP5 and NET expression, and a decrease in CREB, GRIK4, VEGF, ARRB2 expression.
(Sabbagh et al., 2018)	Mice *ex vivo* slice cultures and wild-type primary neurons	dexamethasone	100 nM and 0.5 μM	3 and 4 h	Benztropine increases glucocorticoid-induced GR nuclear translocation in the presence of high levels of FKBP5.
	M17 neuroblastoma	hydrocortisone	50 nM	16 h	CORT induces GR activity and to a lesser extent in the presence of an FKBP5 vector.
(Karst et al., 2000)	Mouse CA1 pyramidal neurons	corticosterone	100 nM	20 min	Exposure of hippocampal neurons to GCs is initiated by the homodimerization, translocation, and GR binding to DNA as seen by an increase in peak and sustained calcium current amplitude.
(Cote-Vélez et al., 2008)	Primary hypothalamic cultures	dexamethasone	10 nM	1 or 3 h	DEX exposure leads to an increase in mRNA expression of TRH upon binding to the GR, through the activity of PKC and ERK signaling.
	SH-SY5Y neuroblastoma	dexamethasone	10 nM	1 or 3 h	
(Diaz‐Gallardo et al., 2010)	Primary hypothalamic cultures	dexamethasone or corticosterone	10 nM or 100 nM	1 h	Cells treated with GCs reveal that several transcription factors including p-CREB, c-Jun, and c-Fos bind to the TRH promoter. This effect was antagonized in the presence of cAMP.
(Pérez-Martínez et al., 1998)	Primary rat hypothalamic cell cultures	dexamethasone	10 nM–10 mM	1–3 h	DEX regulates the expression of *TRH* in a dose-dependent manner, while low and high concentrations inhibit or reduce its expression, intermediate doses provoke an enhanced *TRH* expression.
(Cote-Vélez et al., 2005)	Primary hypothalamic cell cultures	dexamethasone	10 nM	1 or 3 h	DEX exposure provokes interference on the cAMP pathway and upregulates *TRH* expression via CRE and GRE at a transcriptional level.
	SH-SY5Y neuroblastoma	dexamethasone	10 nM	1 h	
(Jeanneteau et al., 2008)	Rat cortical brain slices [P9 and P10]	dexamethasone	1 μM	0.25; 0.5; 2; 4; or 6 h	GCs enhance the activation of TrkB receptor independent of neurotrophins resulting in neuroprotective effects.
	Rat cortical neurons (deprived of B27 for 5 h)	corticosterone	1 μM	3 h	
	Rat cortical and hippocampal neurons	dexamethasone	1 μM	4 h	
(Kumamaru et al., 2008)	Rat hippocampal neurons [P2]	dexamethasone	0.1; 1; 10; 100 μM	3 days	In immature neurons, DEX exposure led to a decrease in BDNF-stimulated dendritic outgrowth and levels of synaptic proteins. In mature neurons, DEX led to a decrease in BDNF-induced postsynaptic calcium influx and presynaptic glutamate release.
(Kumamaru et al., 2011)	Rat cortical neurons [P2]	dexamethasone	0.01–10 μM	4 days	DEX inhibits Sph2-TrkB interaction possibly via suppression of ERK signaling.
(Pandya et al., 2014)	Primary cortical neurons	corticosterone	1 μM	3 or 48 h	Acute GC exposure upregulates the TrkB receptor via activation of the GR in young neurons only. While chronic GC exposure downregulates TrkB expression in both young and mature neurons.
(Numakawa et al., 2009)	Rat cortical neurons	dexamethasone, corticosterone	1 μM	24 or 48 h	DEX and CORT chronic exposure decreased BDNF-mediated release of glutamate via suppression of PLC-γ/Ca^2+^ signaling. Additionally, TrkB-GR interaction was reduced due to a decrease in GR expression.
(Gite et al., 2019)	SH-SY5Y neuroblastoma	corticosterone	500 μM	24 h	CORT exposure decreased viability of neurons, and mRNA expression of BDNF-VI and CREB1.

c. Cellular processes underlying GC effects					
Publication	Cell line/model	Glucocorticoid tested	Concentration(s) used	Exposure duration	Primary finding
(Cruceanu et al., 2022)	Human induced pluripotent stem cell (iPSC)-derived cerebral organoids	dexamethasone	10, 100, and 1000 nM and 100 μM	4 and 12 h	DEX exposure show delayed transcript regulation of differentiation and maturation processes due to GR activity. DEX exposed neurons also display differential expression in genes associated with behavioral phenotypes and disorders.
(Anacker et al., 2013a)	Immortalized human hippocampal progenitor cell line HPC03A/07	cortisol	100 nM and 100 μM	3 days	Low CORT concentrations increased proliferation of progenitor cells and differentiation into astrocytes, and decreased neurogenesis via MR activation. High CORT concentrations decreased proliferation and neurogenesis via GR activation.
(Karst et al., 2005)	Mice hippocampal slices	corticosterone	1–100 nM	0–5 min or 5–10 min or 2.5–50 min	CORT rapidly increases mEPSC frequencies in hippocampal cultures and decreases paired-pulse facilitation. This GC rapid effect is mediated mainly via the MR.
(Munier et al., 2012)	Murine human embryonic stem cell-derived neurons	corticosterone	100 nM	6 h	CORT had differential effects on the Bcl2/Bax ratio in wild-type neurons and neurons with overexpressed MR, with the Bcl2/Bax ratio being substantially increased in the MR-overexpressed neurons.
(Nürnberg et al., 2018)	Human iPSC-derived neural progenitor cells and neurons	dexamethasone	5; 50; 500 nM 50 nM	7, 14, 28, 50 days	DEX exposure leads to an increase in NPC proliferation and a decrease in neuronal differentiation mediated via the GR. The enzyme 11-β-hydroxylase CYP11B1 involved in GC synthesis was expressed in both NPCs and neurons.
(Ninomiya et al., 2014)	iPSC-derived neural progenitor cells and neurons	dexamethasone, bethamethasone, and hydrocortisone	5 and 500 nM and 50 μM	4 days	Different GCs led to an increase in NPC proliferation with increasing concentrations. An increase in MAP2+ neurons was also observed. Under oxidative stress conditions, HDC only led to an increase in MAP2+ neurons.
(Abdanipour et al., 2015)	Rat neural stem/precursor cells from sub-granular and sub-ventricular zones	cortisol	0; 0.25; 0.5; 1; 2.5; 5; 10; 15; 20 μM	24; 48; 72; 96; 120 h	High concentrations of cortisol have anti-proliferative effects on NSCs in a dose- and time- dependent way via apoptosis and necrosis.
(Yao et al., 2007)	Primary rat embryonic hippocampal neurons	dexamethasone	0.01; 0.1; 1; 10 μM	48 h	Exposure to DEX increases susceptibility to the effects of amyloid-β, increases intracellular calcium concentrations, and reduced the amyloid-β-induced expression of NF-κB p65 proteins.
(Koo et al., 2010)	Rat adult hippocampal progenitor cells	corticosterone	10 μM	2 h	CORT negatively affected proliferation of cells with no influence on cell death, This effect is mediated by p39 MAPK signaling and the GR.
(Behl et al., 1995)	Mouse HT22 hippocampal neurons	corticosterone	100 μM	20 h	Exposure to GCs did not show neuroprotective effects in the presence of neurotoxins, leading to a substantial decrease in cell survival.
(Anacker et al., 2013b)	Human hippocampal progenitor cell line HPC03A/07	cortisol	100 μM	1, 3, 12, or 72 h	CORT reduces hippocampal progenitor cell proliferation and differentiation via an increase of *SGK1* expression. Inhibition of Hedgehog signaling and increase of GR function are mediated by SGK1.
(Kim et al., 2004)	Adult rat hippocampal progenitor cells.	dexamethasone	5 μM	12 h	DEX exposure inhibits proliferation of progenitor cells, enhances p21 expression, and impairs ERK activation and SRE activity.
(Yu et al., 2004)	Primary rat fetal hippocampal progenitors	corticosterone and dexamethasone	2, 20, 200 nM/2, 5, 20, 40, 50 μM	3 days	CORT reduces cell proliferation alters *NeuroD*, *BDNF*, and NR1 expression, and provokes dendritic atrophy in a dose-dependent manner.
(Crochemore et al., 2005)	Primary hippocampal rat neurons	dexamethasone	1 and 10 μM	48 h	DEX provokes neuronal cell death via GR-mediated apoptosis
(Tamura et al., 2005)	SH-SY5Y neuroblastoma	corticosterone	0.6 mM	1; 3; 6; 12; 24 h	CORT exposure decreases *Tll-1* promoter activity and *Tll-*1 mRNA expression.
(Anacker et al., 2011)	Human hippocampal progenitor cell line HPC03A/07	dexamethasone and cortisol	1 μM DEX and 100 μM CORT	72 h, 7 days, and 10 days	Antidepressant reverses GC-induced decrease in proliferation and neurogenesis via GR-mechanisms involving PKA signaling, GR phosphorylation, and upregulation of *GADD45B, SGK1,* and *FOXO1* expression.
(Xi et al., 2011)	Primary rat hippocampal neural stem cells	dexamethasone	0.01, 0.1, 0.5, and 1 μM	48 h	Antidepressants reverse DEX-inducing upregulation of TREK-1 and reduction in NSC proliferation.
(Yeo et al., 2019)	SH-SY5Y neuroblastoma and human ESC-derived neural stem cells and neurons	dexamethasone	1; 10; 100; 250; 750; 1000 μg/mL (between 2 μM and 2 mM)	48 h	DEX led to a decrease in cell viability via an increase in apoptosis, and a decrease in pAkt levels.
(Pu et al., 2007)	Rat brain slices	corticosterone	100 nM	15 or 20 min	CORT differentially regulates beta-adrenergic associated synaptic plasticity, depending on the timing of administration.
(Jafari et al., 2012)	Mice adult hippocampal slices	dexamethasone	5 μM	5, 15, or 30 min	DEX exposure modulates synaptic plasticity via alterations in p-Cofilin levels, ERK1/2, number of PSD95+ spines, and pCofilin immunoreactive spines.
	Sprague Dawley rat cultured hippocampal slices	dexamethasone	5 μM	15 min to 1 h	
(Bhargava et al., 2002)	Rat hippocampal H19-7 neurons	corticosterone	100 nM	30; 60; 120 min	CORT leads to an extended increase in intracellular calcium concentrations via the inhibition of PMCA1.
(Suwanjang et al., 2013)	Primary Sprague-Dawley rat cortical midbrain, and hippocampal neurons, and astrocytes	Dexamethasone and corticosterone	1 μM	3–5 min	Brief exposure to GCs reduces basal levels of cytosolic calcium concentrations in both neurons and astrocytes via the GR and independent of the NMDAR, without showing signs of toxicity. These results suggest that GCs are used for the protection of neurons from glutamate cytotoxicity.
(Chen et al., 2011)	Wistar rat hypothalamic primary neuronal slices	Dexamethasone	10 μM	Within seconds to min	Rapid effects of DEX led to a decrease in intracellular calcium concentrations in primary rat hypothalamic neurons. This is suggested to be mediated via GR and plasma membrane calcium pumps activation.
(Du et al., 2009)	Rat primary cortica neurons [E18]	Corticosterone	100 nM, 500 nM, and 1 μM	Ranging between 0 and 72 h	High CORT levels lead to kainic acid induced toxicity and changes in mitochondrial function in cortical neurons, partly via a decrease in GR/Bcl-2 levels in the mitochondria.
(Luo et al., 2021)	Sprague-Dawley rat primary cortical neurons [E18]	Corticosterone	100 nM and 1 μM	30 min, 24 h or 3 days	CORT exposure regulates the formation of GR/Bag-1 complex in a dose and time-dependent manner in rat primary cortical neurons. Prolonged exposure led to a negative regulation of the complex and a reduction in mitochondrial GR levels.
(Zhu et al., 2018)	Mice hippocampal primary neurons (7DIV)	corticosterone	10 μM	24, 48, or 72 h	GCs significantly increase levels of NF-κB subunits, activating NF-κB signaling.
(Bharti et al., 2018)	Mouse HT22 hippocampal neurons and primary cortical neurons	corticosterone	0.5; 1; 2 μM	5 days	Chronic CORT exposure leads to an increase in the Txnip protein expression in both the nucleus and cytosol by activation of the GR. Txnip was also shown to enhance protein nitrosylation and sulfenylation contributing to oxidative damage.
(Seo et al., 2012)	Mouse HT22 hippocampal neurons	corticosterone	200, 400, or 800 ng/mL	24 h	CORT exposure leads to an increase in superoxide levels by upregulating NAPDH oxidase.
	SH-SY5Y neuroblastoma	cortisol	400, or 800 ng/mL	For 2 h daily between 1 and 3 days or 24, 48, 72 h	CORT exposure leads to an increase in superoxide levels by upregulating NAPDH oxidase.
(Iqbal et al., 2015)	SH-SY5Y neuroblastoma	dexamethasone	10 μM	24 h	DEX decreases cell viability and increases endogenous SGK1 expression which carries neuroprotective effects on ROS, mitochondrial dysfunction, and cell death.
(Kim et al., 2018)	SK-N-SH neuroblastoma	corticosterone	0.25 mM	1 h	CORT exposure decreases cell viability, ATP levels, MMP, gene expression of CREB and BDNF. To the contrary CORT increases ROS levels, caspase-3/7 activity, and pro-inflammatory cytokines.
(Golde et al., 2003)	Male Sprague-Dawley rats cortical cultures [E16] and primary microglia and N9 murine microglia cell line	dexamethasone	1; 10; 100; 1000 nM	3 days	DEX exposure leads to the alleviation of neurotoxicity by decreasing NO synthesis and a reduction in iNOS mRNA and protein levels.

d. GC effects on glial cells					
Publication	Cell line/model	Glucocorticoid tested	Concentration(s) used	Exposure duration	Primary finding
(Snijders et al., 2020)	Primary human microglia from post-mortem brain tissue	dexamethasone	1 μM	72 h	DEX exposure promotes the expression of *CD163*, *CD200R* and *MRC1* in microglia. These changes observed are not different between healthy and MDD patients.
(Melief et al., 2012)	Primary human microglia from post-mortem brain tissue	dexamethasone	2 nM	72 h	DEX exposure leads to morphological changes in microglia and upregulates CCL18, CD163, and the mannose receptor.
(Unemura et al., 2012)	Rat primary cortical astrocyte monoculture	corticosterone and dexamethasone	0.01; 0.1; 1 μM	1-6; >12; 24h; and 72 h	GC exposure impairs astrocyte proliferation but not cell death due to GR downregulation via GR activation.
(Crossin et al., 1997)	Rat primary cortical astrocytes	dexamethasone, corticosterone, hydrocortisone	0.1–10 nM; 0.01 and 1 μM	6 h	GCs impairs astrocyte proliferation in a concentration-dependent fashion.
(Virgin et al., 1991)	Rat primary hippocampal astrocytes and secondary hippocampal, cortical, and cerebellar astrocytes	corticosterone, dexamethasone, cortisol	1, 10, 100 nM; 1, 10 μM	24 h	CORT exposure causes an inhibitory dose-dependent effect on glucose transport and increases sensitivity to hypoglycemia, particularly in hippocampal cells.
(Heard et al., 2021)	hiPSC-derived astrocytes	cortisol	5, 50 μM	24 h or 7 days	Chronic exposure to CORT resulted in MDD-specific differentially expressed genes associated with GPCR-ligand binding, synaptic signaling, and ion homeostasis in astrocytes.
(Miguel-Hidalgo et al., 2019)	Rat embryonic myelination neural cultures [E16] and mixed glial rat brain cerebral cortex[P1]	corticosterone	5, 50 μM	4 days (with and without replenishing) or 16 days (replenishing every 3 days)	Chronic exposure to GCs decreases myelination index, MBP and Cx43 in spinal cord and cerebral cortex myelination cultures, that is dose-dependent, mediated by the GR. Additionally, chronic glucocorticoids reduce oligodendrocyte processes.

e. GC effects on neurotransmitter systems					
Publication	Cell line/model	Glucocorticoid tested	Concentration(s) used	Exposure duration	Primary finding
(Groc et al., 2008)	Sprague-Dawley rat hippocampal neurons [E18]	corticosterone	10, 50, 100 nM	1–20 min; 150 min (with washout)	CORT increases hippocampal glutamate transmission in a time-dependent fashion via upregulation of the surface synaptic protein GluR2.
(Zhou et al., 2012)	Rat hippocampal primary cultures	corticosterone	30 nM	15min	CORT in combination with a β-adrenergic receptor agonist regulate AMPAR phosphorylation, surface expression, and mEPSC.
(Mahmoud and Amer, 2014)	Young rat hippocampal slices/tissue	corticosterone	0.5; 5; or 30 nM	1 or 2 h	Brief exposure to CORT is shown to increase synaptic transmission and decrease the NMDAR subunit NR2B and NR2B:NR2A ratio.
(Fan et al., 2018)	SH-SY5Y neuroblastoma	corticosterone	5 μM	3 days	CORT exposure led to an increase in Phox2a and Phox2b via GR activation.
(Pu et al., 2009)	Rat brain slices from the basolateral amygdala	corticosterone	100 nM	20 min–2 h	CORT slowly inhibits synaptic potentiation activated by noradrenergic effects through the β-adrenergic receptor, preventing the system from enhanced activation.
(Wong et al., 2015)	SH-SY5Y neuroblastoma	dexamethasone	100 nM	24 h	DEX exposure upregulates the expression and catalytic activity of MAO A.
(Tazik et al., 2009)	SH-SY5Y neuroblastoma and glioblastoma 1242-MG cells	dexamethasone	10 μM	Every other day for 4 days	DEX exposure impairs cell proliferation and increases the activity of MAO B promoting cell death which could be prevented by antidepressant drugs or MAO inhibitors.
(Johnson and Ou, 2010)	SH-SY5Y neuroblastoma	dexamethasone	2 μM	Daily for 3 days	DEX exposure provokes an increase in the catalytic activity of MAO enzymes leading to cell death and DNA damage, these effects can be counteracted or reduced by MAO inhibitors like M30.

Abbreviations: CORT: corticosterone; CRE: cAMP response element; DEX: dexamethasone; DIV: days *in vitro*; DMS: differentially methylated sites; GC: glucocorticoid; GR: glucocorticoid receptor; GRE: glucocorticoid response element; HDC: hydrocortisone; iPSC: induced-pluripotent stem cell; iNOS: inducible nitric oxide synthase; MAO(-B): monoamine oxidase (-B); MDD: major depressive disorder; MR: mineralocorticoid receptor; NA: noradrenaline; NMDAR: N-methyl-D-aspartate receptors; NO: nitric oxide; NPC: neural progenitor cell; NSC: neural stem cell; PLC-γ: Phospholipase C Gamma; PTSD: port-traumatic stress disorder; ROS: reactive oxygen species; SER: serum response element; TrkB: Tropomyosin receptor kinase B.

## 2 Considerations for GC experiments *in vitro*

*In vitro* experiments investigating the effects of GCs on neuronal cultures (overview can be found in Table 1, and Fig. 4) even though promising, are associated with a number of challenges and limitations. These include a lack of standardized protocols for acute and chronic GC exposure, a broad range of GC concentrations investigated, variability between *in vitro* models, and lack of standardized assessments for GC-induced phenotypes. It is important to consider these aspects when replicating or designing new experiments (Bassil et al., 2022).

### 2.1 *In vitro* definitions of stress

There is a lack of consensus regarding key terminologies such as acute versus chronic and short-term versus long-term effects, which makes it difficult to compare results and interpret them. To improve reproducibility, it is suggested to make use of established acute and chronic stress paradigms in animal stress experiments, tailored to specific types of models (2D versus 3D). For instance, while a 3-day GC exposure in some 2D-neuronal cultures such as induced neurons, can be considered chronic exposure, this duration is not sufficient to investigate chronic effects in cerebral organoids, which have prolonged time windows.

### 2.2 Sources of variability

Sources of variability in GC *in vitro* studies include highly variable concentrations, differences in differentiation protocols and *in vitro* models. GC concentrations used *in vitro* range from as low as 10 nM to 2 mM, including concentrations that do not resemble *in vivo* concentrations. It remains a challenge to accurately measure CORT levels immediately after experiencing a stressor in humans (Baker et al., 2005), even in situations where a better estimation can be made, such as maternal stress (Krontira et al., 2020) and pregnancy (see supplementary information in (Cruceanu et al., 2022)). Importantly, GCs are also known to bind to the plastic of the culture dish (Kelava et al., 2022) and have different half-lives among different types of GCs (Yasir et al., 2018), meaning the final effective concentrations may vary. Interestingly, despite making use of high concentrations (e.g., 1 μM or higher), *in vitro* GC exposure often does not seem to exert profound neuronal effects. This observation could be due to the fact that *in vitro* neuronal cultures are supplemented with a variety of growth and neurotrophic factors, including serum (e.g., SH-SY5Y cultures) or supplements such as B27 (e.g., PSC-derived neurons) which already contains steroids essential for proper neuronal growth and maturation (Viho et al., 2019). The presence of GCs in neuronal media ensures neuronal survival in-a-dish which could minimize the effects of exposure to GCs *in vitro*, hence requiring higher concentrations of GCs to ensure sufficient downstream effects of GR activation (Anacker et al., 2013a). However, this could also be due to the lack of functional GR/MRs in some neuronal cell models (Lieberman et al., 2017) (see Fig. 3 for comparison between models). Differences in culture and differentiation protocols among *in vitro* models, and specifically in reprogramming and differentiation protocols within PSCs (transdifferentiation versus indirect conversion) (Hoffmann et al., 2019) are also major sources of variation (some more than others) that could influence the GC-induced phenotype (as seen in (Seah et al., 2022)). Nevertheless as showcased in Fig. 3, each model (and specifically differentiation protocols) carry advantages and limitations that speak to unique research questions and should be taken into consideration in the selection of the model (Bassil et al., 2022). Given that the generation of iPSCs from donors retain the genotype and in some instances even traces of the epigenotype, iPSC-based models can be a promising for investigating gene-environment interactions (Qian et al., 2016), especially that SRDs cannot be explained by underlying genetic vulnerability alone. This model also has the advantage that a variety of neuronal subtypes can be generated (i.e., dopaminergic, serotonergic and cortical neurons (Hong and Do, 2019; Mertens et al., 2016; Krystal and Neumeister, 2009)), allowing for region-specific GC-induced phenotype identification since maladaptive changes induced by chronic GC exposure in the prefrontal cortex and hippocampus, for instance, reflect an opposite trend as compared to the amygdala (Kim et al., 2015).

### 2.3 Assessment of GC-induced phenotypes

It remains challenging to identify objective and standardized readouts to characterize distinct GC-induced phenotypes. One constant readout that all studies investigating GC effects could include is measuring whether GC treatment does activate the GR and/or MR. For example, measuring the expression of known GC-responsive genes such as FKBP5, TSC22D3, SGK1, ZBTB16, among others. Another major concern is the lack of objective biomarkers for psychiatric disorders, hence the inability to select robust cellular or molecular readouts to characterize specific disease-phenotypes *in vitro* (Falk et al., 2016; Brennand et al., 2014). Current neurobiological models of psychiatric disorders do not capture the full range of clinical manifestations. For instance, no single biological process is present in MDD, and MDD symptoms involve neurobiological circuitries that overlap with other psychiatric disorders (e.g., PTSD) (Van Praag et al., 1990). Nonetheless, a few characteristics to reflect cellular phenotypes of particular psychiatric disorders can be incorporated in *in vitro* studies, which include: cellular phenotypes must (1) match underlying biological pathways; (2) be measurable; and (3) be reversed using pharmacological interventions (Falk et al., 2016). Promising examples include changes in dendritic morphology that can be measured *in vitro* (Forrest et al., 2018), and making use of cell-type associations of key cognitive and psychiatric traits using (Bryois et al., 2020).

Finally, the simplistic/reductionist approach of *in vitro* models will always be an important limitation, as they examine changes occurring within a highly controlled, artificial environment. *In vitro* studies on specific pathways associated with SRDs cannot possibly capture the complexity of stress effects, knowing that the HPA-axis is in active concert with other relevant stress-related processes (Chrousos, 2000). For instance, it is important to note the discrepancy between *in vivo* stress exposure and CORT administration specifically, since changes observed *in vivo* following stress exposure (see example in (Roceri et al., 2004; Mao et al., 2010; Li et al., 2012)), is not the same as administering CORT (see example (Jacobsen and Mørk, 2006)). This observation could be attributed to the fact that the stress response does not only involve GCs but also other hormones and molecular mediators (Fan et al., 2018) such as noradrenergic signaling. Moreover, many studies investigating the effects of DEX, a GR-agonist, in neuronal cell lines cannot reflect the effects of stress or endogenous GCs due to the fact that DEX has a much higher affinity to the GR than other receptors implicated in the stress response such as the MR (Karst et al., 2005). CORT is known to display higher affinity to the MR as compared to the GR (de Kloet, 2014), with MR activation being associated with neuroprotective effects (Munier et al., 2012), and GR activation - in the presence of high GC concentrations - exhibits harmful effects in neuronal cell types (Anacker et al., 2013a). Additionally, MR and GR activation leads to both slow genomic and rapid non-genomic effects that involve a variety of pathways and signaling cascades (Koning et al., 2019; Joëls et al., 2008). This balance between concentration and receptor binding is important in understanding stress vulnerabilities and downstream effects.

## 3 Genetic and epigenetic variations underlying GC effects

Psychiatric disorders are characterized by underlying genetic variants that in combination with environmental stimuli render an individual vulnerable to disease after exposure to factors such as stress (DE KLOET, 2004). For instance, individuals carrying the *FKBP5* rs1360780 risk variant have been documented to be at increased risk of developing psychiatric disorders including schizophrenia and PTSD (Binder et al., 2008; Zannas and Binder, 2014). While genetic variations in *NR3C1, NR3C2*, *CRH*, *CRHR1*, and *BDNF* have also been shown to be involved in conferring risk to SRDs (see reviews (Ebner and Singewald, 2017; Plieger et al., 2018; Chen et al., 2006)), *in vitro* studies in this field have primarily focused on *FKBP5*. Additionally, Arloth et al. (2015) demonstrated that common genetic variants associated with MDD and schizophrenia modify the transcriptional responsiveness of GR target genes (Arloth et al., 2015). Here, we will discuss these *in vitro* studies, which are summarized in Table 1(a).

### 3.1 Genetic vulnerabilities

In recent years, it has become more evident that both genetic and environmental factors interact to confer risk to psychiatric disorders (Chen, 2019; Russo et al., 2012; Sharma et al., 2016). Genetic variants, including single nucleotide polymorphisms (SNPs), are strongly associated with several psychiatric disorders (Committee, 2009), and alter the response of a single individual to particular substances such as drugs, and other environmental stimuli (Matsa et al., 2016). The use of *in vitro* models, and in particular iPSCs, for the investigation of genetic vulnerability of psychiatric disorders has gained increased attention (Seah et al., 2022; Hoffmann et al., 2020), and can be used for understanding how genetic variants create differential cellular responses to, for instance, a GC challenge *in vitro* (Hu et al., 2021).

Looking at the influence of environmental factors in the presence of underlying genetic vulnerability, Seah et al. generated iPSC-derived mixed forebrain neurons and NGN2-induced neurons from combat-exposed veterans with and without PTSD (Seah et al., 2022). Following exposure to different concentrations of DEX, differentially expressed genes were observed for each of the different concentrations in NGN2 neurons and, to a lesser extent, in mixed forebrain neurons. The GC responses on gene expression profiles were enriched for synaptic genes. This is a proof-of-principle study showcasing that the use of stem cell models may facilitate a better understanding of gene-environment interactions in SRDs.

Hay et al. investigated the binding of GR to a highly conserved response element, called 2 GR, within the promoter region of the *TAC1* gene, which codes for the neuropeptide substance-P. This was done in primary rat amygdala cells and in SH-SY5Y cells following acute stimulation with DEX (Hay et al., 2014). An increase in TAC1 was observed following DEX exposure, which was mediated via GR binding to 2 GR within the *TAC1* promoter. A second relevant GR binding site was also identified and designated as SNPGR. SNPGR bears a T-allele polymorphism (found specifically in Japanese and Chinese populations) that enhances the stimulation of the substance-P promoter via the re-activation of the 2 GR subunit. The findings on this polymorphism suggest a genetically underpinned vulnerability to GCs that may be involved in differential GR regulation and homeostasis in health and disease states (Hay et al., 2014), as was also shown by Arloth et al.

Not all studies were successful at demonstrating gene-environment interactions *in vitro*. The availability of iPSC technology has enabled us to investigate the effects of stress (i.e. GC exposure) on human neurons from individuals with an underlying genetic vulnerability for SRDs. One of the first studies attempting this was conducted by Lieberman et al. (2017) who studied changes in mRNA expression of *FKBP5* and *NR3C1* following a 6-h DEX exposure (1 μM) to iPSC-derived cortical neurons from individuals with *FKBP5* rs1360780*C/C and *FKBP5* rs1360780*T-allele carriers. Acute DEX exposure increased mRNA expression of *FKBP5*, but not of *NR3C1*, irrespective of genotype (Lieberman et al., 2017). Nold et al. (2021) exposed mouse primary neuronal cortical and hippocampal cultures, derived from humanized mouse strains carrying either the risk (A/T)or resilient (C/G) allele of rs1360780 of the *FKBP5* locus, to concentrations of DEX ranging from 0.8 to 100 nM for a short incubation time (4 h). While they did not find any significant changes in *NR3C1* expression between different DEX concentrations, they found dose-dependent increases in *FKBP5* expression. Interestingly, no significant effect of the risk versus resilient rs1360780 allele were observed (Nold et al., 2021). Despite both studies not illustrating any effect of the genotype on expression, this is not representative of the field as a whole, with gene-environment interaction being demonstrated in human studies (Klengel et al., 2013; Czamara et al., 2019), and iPSC-derived models (Seah et al., 2022; Dobrindt et al., 2020). Additionally, these studies highlight the importance of cell type differential responsiveness to GCs and GR-sensitivity, but also the importance of *in vitro* studies in unraveling the genetic risk underlying SRDs. This first wave of iPSC-based studies provided several novel insights into the use of *in vitro* studies to infer causation between genetic variance and mechanisms of disease, while also raising many questions which will be addressed in the discussion below.

### 3.2 Epigenetic mechanisms

Epigenetic dysregulation has been associated with a number of disorders including stress-related neurodevelopmental and other psychiatric disorders, as reviewed in (Franklin and Mansuy, 2011; Kubota et al., 2012; McEwen et al., 2012). Some of the long-term effects of GCs may be mediated via epigenetic changes, that are especially pertinent during certain developmental stages (Zannas and Chrousos, 2017; Bartlett et al., 2019). Evidence indicates that GCs can impact epigenetic regulation in two ways: first by moderating the expression of epigenetic regulators and second by inducing epigenetic changes directly at GRE sites (Klengel et al., 2014). For example, genome-wide decreases in DNA methylation levels were observed in proliferating neural stem cell (NSC) cultures *in vitro* following exposure to DEX, which was shown to be mediated via an increased expression of *Tet3*, an enzyme essential for active demethylation in neurons and a crucial player in NSC differentiation (Li et al., 2015).

Similarly, decreases in rat embryonic NSC proliferation and alterations in the expression of genes involved in cellular senescence (upregulation) and mitochondrial functions (downregulation) in NSCs following DEX exposure have been attributed to changes in DNA methylation. Decreases in average levels of genome-wide DNA methylation have been observed together with decreases in the levels of DNA methyltransferases (DNMTs). Interestingly, subsequent experiments indicated that these global changes in epigenetic processes conferred an increased vulnerability to other types of stress (i.e. oxidative stress) *in vitro* in daughter cells which were never directly exposed to DEX (Bose et al., 2010, 2015), revealing a level of epigenetic memory due to GC effects.

Another study used a human hippocampal progenitor cell line to study the immediate and long-lasting effects of DEX on transcriptional and DNA methylation changes during proliferation and differentiation. Provençal & Arloth et al. showed that DEX treatment during the proliferation stage resulted in substantial transcriptional and DNA methylation changes (Provençal et al., 2020). Interestingly, DEX exposure after neuronal differentiation resulted in very minimal changes both at the transcriptional and at the epigenetic level. In addition, the DNA methylation changes observed in neural progenitor cells (NPCs) persisted after a wash-out period to remove DEX and even primed the transcriptional responses to a future GC exposure. These results show that the progenitor stage is a critical neurodevelopmental stage in mediating GC effects and that changes in DNA methylation may persist within regulatory sites, priming transcriptional responses to future GC exposures (Provençal et al., 2020). Therefore, focusing on chronic stress alone is not sufficient in exploring the pathophysiology of SRDs, knowing that acute stress may also carry long-term effects (Provençal et al., 2020; Musazzi et al., 2017).

Another study by Lee et al. investigated the effects of chronic CORT exposure on *FKBP5* DNA methylation and gene expression. They observed that seven days after daily CORT exposure in the HT-22 mouse hippocampal cell line, *FKBP5* gene expression was increased, which was associated with a decrease in DNA methylation at intronic enhancers (Lee et al., 2010). Thus, long-term CORT exposure may decrease methylation and increase expression of *FKBP5*, as well as attenuate GR activation and translocation to the nucleus. Similar findings were reported for DEX exposure in a human hippocampal progenitor cell line (Provençal et al., 2020), i.e. increased mRNA expression and decreased DNA methylation in intronic enhancers. These studies indicate that GCs alter the epigenetic and transcriptional landscape. In addition, they demonstrate that *in vitro* neuronal cultures can be used to study these effects.

## 4 Molecular underpinnings of GC effects

The molecular mechanisms underlying GC effects are complex and involve intricate interactions between the GC receptors and various transcription factors and co-regulators. This section will provide an overview of *in vitro* studies looking into the molecular mechanisms underlying GC effects. The listed studies are summarized in Table 1(b).

### 4.1 Glucocorticoid signaling

#### 4.1.1 Glucocorticoid-related genes

The regulation of glucocorticoid signaling is strongly impacted by molecules within the GR complex, as this receptor requires a number of (co-)chaperone proteins for proper functioning and is regulated by homodimerization (McEwan et al., 2004). One of the primary stress-responsive proteins that have been repeatedly linked to GR activity and stress is FKBP5. FKBP5 is a co-chaperone of the GR, which reduces the receptor's affinity to GCs and its translocation to the nucleus, all features of GR resistance. It has been documented that elevated levels of FKBP5 were associated with increased anxiety and decreased stress coping in rodents. In humans, genetic variants and epigenetic alterations leading to increased FKBP5 have been associated with a number of SRDs including MDD and PTSD (Binder, 2009; Klengel and Binder, 2015; Matosin et al., 2018). For instance, exposure to DEX in SH-SY5Y cells led to time-dependent changes in *FKBP5* mRNA expression following short and long-term incubation (Verjee et al., 2018).

The interaction of FKBP5 and the GR has been proposed as a pharmacological target for SRDs. Indeed, cell culture studies by Sabbagh et al. (2018) showed that pharmacological disruption of the FKBP5/GR complex led to a restoration of effects of DEX on GR activity and its translocation from the cytoplasm to the nucleus in primary neurons and M17 neuroblastoma cells. When studied in *ex vivo* brain slices of aged wild-type mice, DEX exposure led to an increased GR translocation from the cytoplasm to the nucleus. This translocation was also observed (albeit to a lesser extent) in the presence of increased FKBP5 levels (Sabbagh et al., 2018). The important role of this interaction has been corroborated by the effects of the selective FKBP5 antagonist SAFit2 (Hartmann et al., 2015). Together, these results offer a promising avenue to selectively target the FKBP5 complex as a potential therapeutic strategy.

#### 4.1.2 Glucocorticoid receptor functioning

Changes in synaptic plasticity, neuronal activity, and cellular processes such as neuronal viability largely depend on the activation of the GR through GR homodimerization. In hippocampal slices of mutant GR mice, with the mutation preventing GR dimerization, Karst et al. showed that CORT-induced increases in calcium currents are dependent on receptor homodimerization and DNA binding. (Karst et al., 2000).

Downstream GR transcription factors are also important in driving the transcription of key genes with neuromodulatory functions. One study sought to study the effects of DEX on the transcription and synthesis of thyrotropin releasing hormone (TRH), a neuropeptide involved in energy metabolism. In primary hypothalamic cultures, an increase in mRNA expression of TRH is observed following DEX. Inhibition of the PKC and MAPK pathways reversed the DEX-induced effects on *TRH*, which was observed via transcriptional modifications and binding of the GR to composite GRE sites of the *TRH* promoter, particularly at the AP-1 site. These results suggest that PKC or MEK mediate the effects of glucocorticoid signaling on *TRH* transcription by decreasing binding abilities of GR to composite GRE's AP-1 binding site (Cote-Vélez et al., 2008).

#### 4.1.3 GC effects on other neuroendocrine genes

In addition to investigating direct effects of GCs on the HPA-axis alone, the use of *in vitro* studies may also facilitate studying the molecular and cellular functioning of other axes involved in GC responses such as the hypothalamic-pituitary-thyroid (HPT)-axis. The HPT-axis has been repeatedly shown to be involved in SRDs (Fischer et al., 2019; Olff et al., 2006). During the last decades, parts of the HPA- and HPT-axes could only be modeled separately in cell culture models. For example, a series of studies investigated the activation of transcription factors required for the transcription of CRH after GC-activation in hypothalamic neurons. Díaz-Gallardo et al. (2010) observed an increase in TRH mRNA expression following CORT or DEX exposure in rat primary hypothalamic cultures mediated via intracellular GR (Diaz‐Gallardo et al., 2010). In another study, Pérez-Martinez et al. (1998) observed a dose-dependent-biphasic response in primary rat hypothalamic neuronal cultures shortly after exposure to DEX. These findings indicate that low concentrations of DEX (0.1 nM) suppressed TRH mRNA expression, while intermediate concentrations of DEX induced an increase of TRH mRNA, and higher levels (1 μM) were associated with decreased expression. Together these results suggest rapid regulatory effects of DEX on TRH mRNA expression in hypothalamic neurons *in vitro* (Pérez-Martínez et al., 1998). This and other studies (Pérez-Martínez et al., 1998; Cote-Vélez et al., 2005; Maroder et al., 1993) investigating GC-stimulated expression of other HPT hormones, including TRH, provide important insights into the interplay between neuroendocrine axes, such as the effects of GCs on *TRH* expression and noradrenaline in stress conditions.

### 4.2 Brain-derived neurotrophic factor

Brain-derived neurotrophic factor (BDNF) plays a crucial role in neuronal processes including neuronal survival and synaptic plasticity (Patapoutian and Reichardt, 2001; Bibel and Barde, 2000). These effects are initiated by the activation of the tropomyosin receptor kinase B (TrkB) receptor and its downstream signaling constituents, including phosphatidylinositol-3-kinase (PI3K), phospholipase Cγ (PLCγ), and MAPK pathways (Murphy and Blenis, 2006), eventually leading to the transcription of relevant genes necessary for survival and plasticity. There is evidence that GCs modulate BDNF signaling. For instance, one study focused on the acute neuroprotective effects of GCs (1 μM) in rodent brain slices (Jeanneteau et al., 2008) and showing that GCs activate TrkB receptors in neurons independently of neurotrophin release (Jeanneteau et al., 2008), which eventually enhanced neuronal survival. This suggests that GCs carry trophic properties by acting on TrkB receptors and induction of a non-canonical Akt signaling pathway.

Kumamaru et al. (2008) showed that DEX exposure in young primary hippocampal neurons reduced BDNF-induced enhancing effects on synaptic plasticity as measured by outgrowth of dendrites and expression of (pre-)synaptic proteins (Kumamaru et al., 2008). DEX also decreased the BDNF-induced MAPK/ERK pathway, which mediates the downstream expression of BDNF-induced genes on survival and synaptic maturation. The effects of DEX on components of the MAPK/ERK pathway activation was further investigated with a focus on Src homology-2 domain containing phosphatase 2 (Shp2). Long-lasting ERK signaling is required for the transcription of BDNF-induced synaptic proteins. Activation of this pathway requires the interaction of Shp2 – an ERK signaling mediator – with TrkB. In the presence of DEX, a reduction in Sph2-TrkB interaction (which is required for ERK pathway activation) was observed suppressing the expression of BDNF-induced synaptic proteins in cortical cultures (Kumamaru et al., 2011).

Another study investigated the acute and chronic effects of CORT on TrkB expression in young and mature neurons derived from primary mouse cortical neurons. Following acute CORT exposure, an increase in TrkB protein levels was observed in early primary cortical neurons but not in mature neurons derived from the same primary cortical cells. Subsequent experiments indicated that this increase may be mediated via c-Cbl, which was shown to co-precipitate with TrkB in the presence of CORT. This CORT-induced increase in TrkB activation was prevented when c-Cbl was knocked down. Following chronic CORT exposure, a significant decrease in TrkB levels was observed in both early and mature cortical neurons. Interestingly, *c-Cbl* mRNA levels have been found to be decreased in both the frontal cortex of mice subjected chronic stress and in the prefrontal cortex of human suicide subjects (Pandya et al., 2014).

Numakawa et al. (2009) demonstrated that chronic (24 or 48 h) exposure to DEX decreased BDNF-mediated release of glutamate via inhibition/suppression of PLC-γ/Ca^2+^-signaling in rat cortical neurons. In addition, the interaction between TrkB and the GR was also reduced following both DEX and CORT exposure, and GR expression was decreased. Interestingly, following *in vitro* siRNA silencing of the GR, the inhibitory/suppression effects of DEX on PLC-γ/Ca^2+^-signaling were replicated while the opposite was observed following GR overexpression. These results suggest the importance of TrkB-GR interaction in the face of BDNF-induced PLC-γ activation needed for the release of the neurotransmitter glutamate (Numakawa et al., 2009).

To investigate whether antidepressants or nutraceuticals (i.e. alternative products derived from herbs and dietary supplements sometimes used for medicinal purposes (Nasri et al., 2014)) can counteract the effects of GCs on cell viability and neuronal plasticity, Gite et al. (2019) used SH-SY5Y cultures. They observed a reduction in cell viability following CORT exposure, in addition to a reduction in mRNA expression of cAMP-responsive element binding protein (CREB)1 and BDNF-VI, both mediating neuronal survival and synaptic plasticity (Gite et al., 2019). These effects were shown to be reversed following addition of antidepressants and a few selected extracts.

## 5 Cellular processes underlying GC effects

As explained above, genetic and epigenetic processes underly molecular mechanisms of GC-induced effects in relation to SRDs. The altered molecular processes can manifest in affected cellular processes too. These may include neurogenesis, synaptic plasticity and neuronal activity, all processes that have been implicated in SRDs. See Table 1(c) for a summary of the listed studies.

### 5.1 Neurogenesis

The formation of new and functional neurons from their precursors is referred to as neurogenesis (Ming and Song, 2011). Neurogenesis mainly takes place during early development, although the existence of adult neurogenesis has been firmly established in rodents, while still debated in human. Mechanisms underlying neurogenesis have been extensively studied using *in vitro* neuronal models, by looking at proliferation, differentiation, cell death and survival (Kuhn, 2015).

The process of neurogenesis is influenced by many factors including hormonal exposure. CORT, for example, has been shown to influence the number of proliferating NSCs and their survival (Tea et al., 2019). While data on CORT affecting proliferation indicates both increased as well as decreased proliferation, the overall impact of CORT on neurogenesis seems to be a reduction in the number of differentiated and functional new neurons, likely through priming of cells for gliogenesis (Nürnberg et al., 2018; Ninomiya et al., 2014; Guo et al., 2009).

There is a lack of consensus on the impact of GCs on neuronal physiology. While some studies report a decrease in viability of NSCs (mainly via apoptotic pathways) with increasing concentrations of GCs (Abdanipour et al., 2015; Yao et al., 2007), one study observed no change in cytotoxicity and cell survival of HT22 mouse hippocampal neuronal cultures with even higher concentrations of CORT (Behl et al., 1995) compared to the aforementioned studies. In contrast, increases in neural progenitor proliferation have also been documented *in vitro* following GR activation. For instance, Anacker et al. observed increased proliferation (as shown by Bromodeoxyuridine (BrdU) staining) and astrogliogenesis, and decreased neurogenesis (MAP2-positive and DCX-positive cells) following low CORT concentrations (100 nM) in immortalized human hippocampal progenitors. High concentrations (100 μM) however, led to decreased proliferation and differentiation (replicated in (Anacker et al., 2013b) in human hippocampal progenitors). The effects of low CORT concentrations were mediated by the activity of the MR, while the effects of high CORT concentrations seemed to be mediated by GR activity, as demonstrated by co-incubation with receptor antagonists. The underlying molecular pathways which were impacted by CORT exposure involved Notch/Hes-signaling in conditions with low CORT concentrations, and TGFβ-SMAD2/3 signaling with high CORT concentrations (Anacker et al., 2013a). A decrease in proliferation but not differentiation has also been reported in adult rat hippocampal progenitors following a 5 μM concentration of DEX (Kim et al., 2004; Yu et al., 2004). In Yu et al. (2004), CORT exposure (2 μM) in fetal hippocampal progenitor cells led to a decrease in both proliferation and differentiation.

MR is highly expressed in the brain, particularly in the hippocampus, and, together with GR, plays a crucial role in neuronal survival (Gass et al., 2000). In line with *in vivo* findings*, in vitro* studies have demonstrated that MR activation and overexpression reverses GC-induced hippocampal neuronal apoptosis via the GR (Munier et al., 2012; Crochemore et al., 2005). These studies highlight the importance of MR activity in stimulating neuronal survival in the presence of GCs. It is important to mention that these neurotoxic effects are most often seen in the presence of high GC concentrations. That being said, identifying the target receptor of interest (GR, MR, or both), which will inform the selection of non-synthetic or synthetic GCs (e.g., DEX for GR or aldosterone for MR), and eventually GC concentration are crucial parameters in drawing conclusions on the effects of GCs *in vitro* and will be discussed further below.

Downstream transcription factors are required for the synthesis of proteins and growth factors involved in neurogenesis. For example, a study by Tamura et al. (2005) looked at changes in mRNA expression of Tolloid-like 1 (*Tll-1*) – a metal-based protease enzyme – whose function is required for the synthesis and functioning of bone morphogenetic proteins (BMPs) required for neurogenesis in the hippocampus of adult mammals. Following exposure to CORT, a decrease in *Tll-1* promoter activity was observed in cultured SH-SY5Y cells. Additionally, this decrease was also associated with a reduction in endogenous mRNA levels of *Tll-1*. Together, these *in vitro* results suggest a role of Tll-1 in modulating neurogenesis *in vivo* in the presence of a stress stimulus (Tamura et al., 2005).

A stress-induced decrease in neurogenesis has been proposed as a possible underlying mechanism for the observed hippocampal atrophy in patients suffering from SRDs such as MDD and PTSD (Sheline, 2000; DeCarolis and Eisch, 2010). Antidepressants for instance, have been shown to reverse stress-induced hippocampal volume reduction in both animals and humans (Vermetten et al., 2003; Malberg, 2004). *In vitro,* antidepressants have also been shown to reverse the GC-induced decrease in neurogenesis (Anacker et al., 2011; Xi et al., 2011).

The use of stem cell technology allows the investigation of the effects of GC exposures not only on proliferating progenitors but also on post-mitotic neurons *in vitro*. For instance, a decrease in viability was observed in a study using human ESC-derived NSCs and differentiated SH-SY5Y cultures. Higher concentrations (100 μM) of DEX led to a decrease in proliferation (as assessed by BrdU) of hESC-derived NSCs, a decrease in the percentage of cells bearing neurites, and an increase in apoptosis (Yeo et al., 2019). Conversely, lower concentrations of DEX (50 nM) induced NSC proliferation and decreased differentiation of human iPSC-derived neurons (Nürnberg et al., 2018). Similarly, DEX (50 μM) and CORT (at varying concentrations) also induced proliferation in human iPSC-derived NPCs. Under oxidative stress conditions, CORT alone, but not DEX, promoted proliferation. The authors concluded that these results highlight the importance of MR activation in conferring the neuroprotective effects during cellular stress conditions (Ninomiya et al., 2014). This further illustrates the differential effects of the MR when compared to GR, with increased MR activity being associated with protective effects in the brain, whereas decreased activity linked to psychiatric disorders (Munier et al., 2012).

Prenatal stress and early exposure to chronic stress have been proposed to increase risk of neurodevelopmental disorders in humans (O'donnell et al., 2009). There is evidence of parallel effects of increased prenatal GC signaling and prenatal stress (Krontira et al., 2020), although the exact link might not be straightforward. To better understand the effects of GCs on neuronal development, a recent study exposed human iPSC-derived cerebral organoids to DEX (100 nM) for an acute period of 12 h and observed a non-cell-type specific expression and activation pattern of *NR3C1*. DEX resulted in an increased transcriptional response of GR-regulated transcripts, such as *FKBP5*, and an accumulation of GR in the nucleus, indicating that DEX activated GR-signaling in cerebral organoids. An increase in *PAX6* in both neural progenitors and neuronal clusters suggests increased proliferation of both progenitor cells and an increase in immature neurons (Cruceanu et al., 2022). Many of the differential expressed genes are known to play a crucial role during neuronal development by regulating neuronal proliferation and safeguarding the neural progenitor pools (Lin et al., 2017; Mi et al., 2013; Nam et al., 2016; Wang et al., 2014). The acute exposure (12 h) was not sufficient to lead to changes in cell number but was able to prime the cells transcriptionally for altered developmental milestones. Their findings validate previous *in vitro* studies showcasing effects of prolonged GR activation on neurogenesis, and neuronal maturation (Provençal et al., 2020). Additionally, DEX-induced gene expression changes within neurons alone were shown to be associated with certain brain behavioral phenotypes and risk for psychiatric phenotypes including MDD, neuroticism, openness, sleep-associated behaviors, intellectual disability, and autism spectrum disorder. Thus, this *in vitro* model is a great first step forward and may serve as a proof-of-concept for the use of increasingly complex *in vitro* human cell models such as 3D cerebral organoids (and maybe one day assembloids of hypothalamic, pituitary, and adrenal organoids) in order to enhance our biological understanding of gene-environment interactions. Even though they do not include vasculature and supporting glial cells, they are characterized by a cytoarchitecture and a heterogeneous population of NPCs (which is seldom considered) that highly resembles *in vivo* conditions.

### 5.2 Synaptic plasticity

Following the generation of a new neuron, synapse formation is one of the next crucial steps in neurodevelopment (Kuhn, 2015). Synaptic plasticity is a physiological process where defined patterns of neural activity lead to long lasting alterations in synaptic functioning and neural excitability. This basic process underlies fundamental functional abilities of the brain such as information storage, and brings about changes in complex behaviors (Martin et al., 2000). Conditions of stress have been shown to impact synaptic plasticity, long-term potentiation (LTP), synaptic potentials, and neuronal activity.

### 5.3 LTP

Impairment of LTP – an increase in synaptic strength – has been observed in adult mice following acute stress (Christoffel et al., 2011). For example, negative effects of CORT on LTP have been shown to be dependent on GABA_A_ receptor blockage and β-adrenergic activation, as seen in an *ex-vivo* study looking at rapid effects of GCs in the hippocampus (Pu et al., 2007).

GC-induced changes in LTP have also been linked to GR expression in hippocampal dendritic spines. Acute exposure to DEX in hippocampal slices led to an increase in phosphorylated (p)-Cofilin and extracellular signal-regulated kinase (ERK)1/2, which is known to play a role in the regulation and stabilization of cytoskeleton actin filaments in spines. Paradoxically, a reduction of (p)-Cofilin levels in spines was also observed after DEX exposure. Together, these results highlight the role of GR in hippocampal dendritic spine function and in the local effects of DEX on synaptic plasticity, specifically on spine actin remodeling (Jafari et al., 2012).

#### 5.3.1 Neuronal activity

CORT has been shown to cause rapid changes in hippocampal activity, by increasing the rate of miniature excitatory postsynaptic potentials (mEPSPs) which can modulate presynaptic properties, trigger an action potential, and eventually lead to glutamate release (Karst et al., 2005). These rapid effects of CORT seem predominantly mediated via the MR and not the GR, causing initial non-genomic changes that are later manifested through genomic signaling pathways. This study highlights MR-GR interplay and indicates a role for MR as a “cortico-sensor” enabling fast non-genomic responses to CORT. Once the MR effects have returned to baseline, it is followed by GR-mediated genomic downstream alterations, illustrating the dual mechanism of CORT leading to both short and long-term changes in hippocampal activity in response to stress.

Well-regulated intracellular Ca^2+^ dynamics are essential for neuronal survival, synaptic plasticity and function (Stevens et al., 2003). A chronic exposure to GCs leading to increased levels of intracellular Ca^2+^ negatively impacts neuronal survival and plasticity (Sapolsky, 2000; McEwen, 1999). Therefore, several studies have investigated the effects of GCs on Ca^2+^ influx in neurons *in vitro* (Kumamaru et al., 2008; Yao et al., 2007; Bhargava et al., 2002; Landfield and Pitler, 1984). One study noted that DEX enhances the toxic effects of amyloid β-protein-induced increases in neuronal CA^2+^ influx. Interestingly, DEX alone had no effect on Ca^2+^ influx in hippocampal neurons following a 24 h exposure (Yao et al., 2007). Bhargava et al. (2002) investigated the effects of GCs on Ca^2+^ transients in hippocampal-derived H19-7 neurons and demonstrated that GCs inhibit the plasma membrane protein Ca^2+^ - ATPase-1 (PMCA1) in these hippocampal cultures, which is needed for detecting intracellular Ca^2+^ levels in neurons. Following CORT exposure, an increase in Ca^2+^ transients was observed in hippocampal-derived H19-7 neuronal cultures, independent of calcium channel activation (Bhargava et al., 2002). Another study observed a decrease in basal Ca^2+^levels in rat cortical neurons following DEX or CORT exposure, or physiological and pathological levels of glutamate (Suwanjang et al., 2013). Chen et al. (2011), demonstrated a reduction in intracellular Ca^2+^ concentration, via Ca^2+^ pumps following high concentrations of DEX in primary rat hypothalamic neurons (Chen et al., 2011). Kumamaru et al. (2008) also investigated the effects of DEX on Ca^2+^ influx and observed a decrease of post-synaptic Ca^2+^ influx induced by BDNF (Kumamaru et al., 2008). Together, these results highlight the role of GCs in regulating Ca^2+^ levels that are required to ensure proper neuronal functioning, calling for increased studies into this mechanism.

### 5.4 Mitochondrial function

GCs have also been shown to play a role in regulating the functioning of mitochondria, which are responsible for generating energy in cells. The mitochondrion for instance is important in facilitating adaptation to stress. Particularly, GCs can inhibit the activity of enzymes involved in the mitochondrial electron transport chain, and even increase levels of mitochondrial reactive oxygen species (ROS) (Picard et al., 2018). Du et al. tested low and high doses of CORT exposure in primary cortical neurons on mitochondrial function. While low concentrations showed neuroprotective effects, higher concentrations led to neurotoxicity through increased levels of kainic acid. The mechanisms of action of high doses of CORT in cortical neurons was shown to include a decrease in the GR/Bcl-2 complex translocation into the mitochondria following acute treatment. Prolonged high CORT treatment however, led to a decrease in GR and Bcl-2 expression (Du et al., 2009). Another study by Luo et al. investigated the effects of CORT on a Bcl-2 associated protein, Bag-1 (Bcl-2 associated athanogene), in GR translocation into the mitochondria. Acute and high concentrations of CORT increased the generation and translocation of the GR/Bag-1 complex into the mitochondria in primary cortical neurons. Bag-1 was demonstrated to regulate GR translocation, with increased expression of Bag-1 inhibiting mitochondrial GR levels following prolonged and high CORT concentrations (Luo et al., 2021). Together these results suggest a concentration- and exposure-dependent response of GCs on mitochondrial function, neuronal survival, and GR mitochondrial translocation. This has further implications for the role of mitochondrial function in conferring resilience or susceptibility to GC challenges in neurons, highlighting that mitochondrial-associated pathways might be potential therapeutic targets for psychiatric disorders (Kokkinopoulou and Moutsatsou, 2021).

### 5.5 Neurotoxicity

Inflammation and the activation of inflammatory signalling pathways, in part due to increases in circulating cytokines, have been related to stress and SRDs (Liu et al., 2017a). A range of cellular studies have provided evidence that NF-κB transcription has an effect on several neuronal processes including proliferation, maturation, and neurogenesis in the presence of stress (Koo et al., 2010). In one *in vitro* study, a single exposure to DEX before the addition of amyloid β fragment 25–35 increased the vulnerability of hippocampal neurons to the inflammation-inducing effects of amyloid β by increasing intracellular calcium levels, and decreasing nuclear levels of NF-κB (Yao et al., 2007; Zhu et al., 2018). Another study on the effects of GCs on NF-κB expression in hippocampal neurons, reported an increase in protein expression of several NF-κB subunits including p50, p56, p-p65 and A-p65 after exposure to CORT for 48 and 72 h (Zhu et al., 2018). These *in vitro* findings indicate that GCs induce an increase in NF-κB transcriptional activity in the hippocampus, which in turn carries anxiogenic properties.

Evidence reflecting oxidative damage has been documented in rodent models of chronic stress. Bharti et al. (2018) exposed HT22 mouse hippocampal neurons and primary cortical neurons to CORT and investigated Thioredoxin (Trx), a protein involved in regulating oxidative protein cysteine changes. While no changes in protein levels of Trx and its reduced form were observed following chronic CORT exposure, a substantial increase in the endogenous Trx inhibitor, Txinp, was observed. Interestingly, this was reversed in the presence of the GR antagonist RU486, also known as mifepristone (Bharti et al., 2018).

In another study using SH-SY5Y cell cultures, CORT treatments led to an increase in levels via upregulation of nicotinamide adenine dinucleotide phosphate (NAPDH) oxidase and induced the production of ROS. These effects were reversed in the presence of NAPDH oxidase inhibitors which suggests an underlying mechanism of SRDs, particular MDD, with NAPDG oxidase inhibition being a potential therapeutic target to pursue (Seo et al., 2012).

Loss of neurons in the CNS is a neuropathological hallmark of neurodegenerative disorders, and can be mediated via inflammatory mechanisms (Lupien et al., 2016). Elevated levels and recurrent exposure to GCs are known to induce neurotoxicity. Kim et al. (2018) acutely exposed human SK-N-SH neuroblastoma cells to high concentrations of CORT. Following CORT exposure, they observed a reduction in cell viability (also reported in another study using SH-SY5Y cells (Iqbal et al., 2015)) and in cellular ATP levels linked with an increase of caspase-3/7 activity – early markers of apoptosis. Mitochondrial function was also impaired via decreased mitochondrial membrane potential, and levels of ROS were increased, including mitochondrial superoxide (Kim et al., 2018).

Other studies have demonstrated anti-inflammatory effects of DEX in rat embryonic cortical neurons co-cultured with microglia stimulated with interferon-gamma and lipopolysaccharide. DEX exposure was shown to downregulate the expression of nitric oxide and inducible nitric oxide synthase produced by microglia, which are known to be neurotoxic to neurons when present in high levels (Golde et al., 2003). That being said, studies into the neurotoxic effects of GCs remain controversial and highly dependent on several conditions such as exposure time, intensity of stimulus amongst others.

## 6 GC effects on glial cells

Although the previous section focused on GC effects on neurons, an increasing number of studies are highlighting the roles of glial cells in SRDs and their involvement in GC effects. Glial cells including astrocytes, oligodendrocytes, and microglia play essential roles in the regulation, support, and protection of neurons (Jäkel and Dimou, 2017). A summary of the listed studies can be found in Table 1(d).

### 6.1 Microglia

Microglia dysregulation has been suggested to be an underlying cause of immune dysregulation seen in MDD patients (Liu et al., 2017b). Changes in microglia activation, morphology and in the level of activation markers have been reported in post-mortem brain samples of subjects with MDD (Enache et al., 2019). One particular *ex vivo* study by Snijders et al. investigated responsiveness of microglia taken from post-mortem brain tissue of MDD patients to GCs. Following a 72-h exposure to DEX, an increase in *CD163* and *MRC1* expression (‘anti-inflammatory’ response genes) was observed, with no change in microglia activation markers. These results suggest that GC-induced microglia responsiveness is affected in patients with MDD (Snijders et al., 2020; Melief et al., 2012).

### 6.2 Astrocytes

Astrocytes play a critical role in regulating the neuronal environment. Recent cell culture studies using primary cortical astrocytes reported that exposure to CORT or DEX was associated with a reduced proliferation of astrocytes which may be mediated via downregulation of GR expression (Unemura et al., 2012; Crossin et al., 1997), and decreased glucose transport and affinity of glutamate uptake in astrocytes (Virgin et al., 1991).

More recently, MDD patient-derived astrocytes were generated from iPSCs and exposed to CORT. Unique transcriptomic responses were observed following acute (24 h) and chronic (7 days) treatment with CORT. Subsequent whole transcriptomic sequencing identified a unique expression profile following chronic CORT in MDD patient-derived astrocytes, with the differentially expressed genes being associated with synaptic signaling, ion homeostasis, and GPCR ligand binding (Heard et al., 2021). These studies highlight the unique effects of GCs in astrocytes, specifically in MDD patients, and offer several opportunities for future research looking into the role of astrocytes in inferring risk for SRDs.

### 6.3 Oligodendrocytes

Myelination, a process driven by oligodendrocytes, is vital for the healthy functioning of neurons. In a recent study, the effects of prolonged exposure to both CORT and DEX were investigated on changes in morphology and immunoreactivity of oligodendrocytes and astrocyte-associated proteins (Miguel-Hidalgo et al., 2019). This study reported a dose-dependent decrease in the co-localization between myelin basic protein (MBP) and phosphorylated neurofilament, termed the myelination index, in spinal cord- and cortical myelinating neuronal cultures. This study reported a decrease in immunoreactivity of MBP and of connexin-43 in both rat embryonic spinal cord and cerebral cortex primary cultures (in both glial cultures and glia-neuron co-cultures) after prolonged exposure to GCs. These effects were prevented by the GR antagonist RU486. These results indicate the toxic effects of CORT on myelin formation *in vitro,* partially mediated via the GR.

Together these results highlight the importance of glial cells in conferring susceptibility to GCs *in vitro.* More studies looking into the interplay of glial cells and neurons via co-cultures in response to GC stimulation, will shed light on how both cell types interact to confer GC-induced effects on vital neuronal functioning.

## 7 GC effects on neurotransmitter systems

Effects of GCs on neurotransmitter systems are complex and involve multiple levels of regulation, including modulationg of gene expression, protein synthesis, and neurotransmitter system release and uptake. This section will provide an overview on *in vitro* studies investigating effects of GCs on neurotransmitter signaling systems. A summary of the listed studies can be found in Table 1(e).

### 7.1 Glutamatergic signaling

Changes in glutamate transmission and release have been observed following exposure to GCs *in vivo* and *in vitro* (Lowy et al., 1993; Popoli et al., 2012)*.* Alpha-amino-3-hydroxy-5-methyl-4-isoxazolepropionic acid receptor (AMPAR) trafficking is essential for the transmission of fast excitatory synaptic activity in the brain. AMPAR trafficking may modulate synaptic plasticity, where increased membrane recruitment of the receptor leads to synaptic potentiation, and increased receptor endocytosis leads to synaptic depression (Anggono and Huganir, 2012). Two studies investigated the effects of CORT alone (Groc et al., 2008) or in combination with the β1-and β2-adrenergic receptor agonist isoproterenol, which facilitates synaptic potentiation (Zhou et al., 2012), on AMPAR activity and trafficking in both *in vitro* primary hippocampal neurons and *ex-vivo* rat coronal brain slices. Short-term CORT (but not isoproterenol) induced increased AMPAR glutamate receptor 2 (GLuR2; a AMPAR subunit) surface mobility and synaptic surface expression exclusively via the activity of MRs, which eventually facilitates potentiation. However, in the long-term, CORT slowly increased surface GluR2 and trafficking, exclusively via the activity of GRs, leading to impeding synaptic potentiation (Groc et al., 2008). Hence, this CORT-induced increase in AMPAR surface trafficking is time- and receptor-dependent and carries consequences on the regulation of synaptic plasticity. Zhou et al. showed that CORT alone had no effect on AMPAR phosphorylation, surface expression of GluA1 and GluA2 or miniature excitatory postsynaptic currents (mEPSCs). However, increased AMPAR phosphorylation, GluA1 and GluA2 expression, and decreased inter-event interval of mEPSCs was seen when isoproterenol and CORT were combined together (Zhou et al., 2012). These results highlight the interaction between GC and adrenergic signaling on glutamate transmission.

Besides AMPA-signaling, glutamatergic transmission is also affected by the expression and functioning of the N-methyl-D-aspartate receptor (NMDAR). Mahmoud and Amer (2014) investigated the effect of GC exposure on hippocampal activity by investigating the effects of CORT on the protein expression of NDMAR subunits NR1, NR2B, and NR2A (Mahmoud and Amer, 2014). The protein levels of these subunits were decreased following exposure to low dosages of CORT, which was reversed in the presence of growth hormone (GH), highlighting how GC-induced effects on synaptic transmission are reversed in the presence of low doses of GH. This study highlights that GC-induced effects involve the inhibition of neuronal processes via NMDAR activity (Nacher and McEwen, 2006).

### 7.2 Noradrenergic functioning

The noradrenergic system in the brain is one of the key players and regulators of the stress response together with glucocorticoid signaling. Interestingly, it is implicated in stress-related affective disorders such as MDD and PTSD (Itoi and Sugimoto, 2010). Noradrenergic mechanisms, which involve norepinephrine (NE), play a key role in the process of fear conditioning and in the development of PTSD (Hendrickson and Raskind, 2016). The mechanisms involved in fear conditioning are suggested to be mediated by the release of NE in the amygdala, strengthening the experience of fear conditioning. In PTSD, the process of fear conditioning is dysregulated, and it has been suggested that the noradrenergic system is overactive, leading to an exaggerated fear response, a key symptom associated with PTSD (Southwick et al., 1993).

To investigate the effects of stress and GCs on noradrenergic functioning, Fan et al. (2018) investigated the effects of CORT in SH-SY5Y cells on the expression of *Phox2a* and *Phox2b* – two homeodomain transcription factors – that are crucial in the development of noradrenergic neurons during embryonic development. Increased expression of these two transcription factors was observed following exposure to CORT as a stressor (Fan et al., 2018). Moreover, in *ex-vivo* rat brain slices and within the basolateral amygdala, CORT reversed the LTP-inducing effects of isoproterenol. This suggests that GCs can reverse the effects of β-adrenergic signaling (Pu et al., 2009).

### 7.3 Serotonergic system

Aberrant functioning of the brain serotonergic system has been associated with SRDs like MDD and documented in human (Smith et al., 1997), animal (Wong et al., 2015), and *in vitro* studies (Vadodaria et al., 2019). Serotonin levels increase following stress, which has a modulatory effect on the functioning of the HPA-axis, limiting the negative consequences of a prolonged activation on inflammation and oxidative stress (Leonard, 2005b). Chronic exposure to stress can in contrary lead to decreased levels of serotonin, which is associated with the development of MDD symptoms, namely changes in mood, sleep patterns, and appetite (Mann, 1999). Antidepressants such as selective serotonin reuptake inhibitors (SSRIs), work on this system by increasing the levels of serotonin in the brain, and as such reversing the symptoms of MDD (Vahid-Ansari and Albert, 2021).

Monoamine oxidase (MAO) is an enzyme responsible for the breakdown of neurotransmitters including serotonin, and inhibitors of MAO are widely used as antidepressants (Volz and Gleiter, 1998). *In vitro*, increased expression of two MAO isoforms, MAO A (Wong et al., 2015; Ou et al., 2006) and MAO B (Tazik et al., 2009), is observed following exposure to DEX. Some studies furthermore demonstrate the inhibitory effects of MAO inhibitors on DEX-induced increased MAO catalytic activity (Tazik et al., 2009), apoptosis and a decrease in cell survival (Johnson and Ou, 2010).

## 8 Conclusion and future considerations

We provide an extensive overview of *in vitro* research findings (Fig. 4) on the effects of GCs on different types of neuronal cultures. It has become clear that *in vitro* studies aid in unraveling the multiple GC-induced cellular and molecular pathways implicated in SRDs. Advances in stem cell technology opens avenues for the investigation of gene-environment interactions that is fundamental in understanding the pleiotropic risk to develop SRDs. GC effects differ across central nervous system cell types (i.e., neuronal subtypes) and depending on whether GC treatment is acute or chronic. It is apparent that *in vitro* studies are split into separate categories. While many studies aim to investigate the underlying mechanisms of GCs, others make use of *in vitro* studies as a validation for *in vivo* findings, and a smaller number of studies aim to test the neuroprotective effects of drugs or nutritional supplements on GC-induced toxicity. As the literature shows a great diversity in experimental conditions, it is not surprising that results remain conflicting.

Nevertheless, *in vitro* neuronal models (especially stem cell-based models) are increasingly showing relevance and promise not only in investigating effects of GC exposure which would allow us to unravel mechanisms underlying stress susceptibility and resilience, but also in their validity in translational clinical efforts, such as the identification of biomarkers (Provençal et al., 2020), close to identifying potential novel therapeutic targets. Therefore, tackling the challenges and limitations that come with *in vitro* setups to investigate effects of GCs is instrumental in order to better understand biological processes moderating and/or mediating the onset and course of SRDs. More elaborate systematic reviews or meta-analyses should be conducted including the different conditions and parameters such as exposure time, concentration range, cell line, and age of culture, to provide a more accurate representation of GC effects *in vitro*. Advances in stem cell technology such as 2D and 3D patient-specific generation of neuronal and glial cultures are expected to help gain new knowledge about individual mechanisms contributing to disease that cannot be understood with human or animal studies alone. Therefore, improved standardized GC paradigms *in vitro* that better reflect *in vivo* conditions during stress could provide useful insights to apply in advanced and complex culture models (Bassil et al., 2022). A few suggested steps to take could include: (1) selecting the appropriate model based on its characteristics (see Fig. 3 for reference) and its potential to answer the research question; (2) selecting the model based on GR/MR expression and model responsiveness to GCs, (3) defining whether acute or chronic exposure is more appropriate, and (4) defining parameters and conditions including concentrations, exposure time, and culture conditions such as the use of culture media in the presence or absence of certain factors with masking effects (e.g., growth factors). For instance, making use of concentrations that are more representative of *in vivo* conditions (as explained in (Du et al., 2009)) would aid in establishing the much needed standardization of *in vitro* studies investigating GC-associated mechanisms. Finally, the question of how best to study the effects of two or more stress mediators together (e.g., NE and CORT) is particularly important and highlights another important challenge that needs to be addressed in future studies.

## Declaration of competing interest

The authors have no conflicts of interest to declare that are relevant to the content of this article.
